# Root Membrane Replantation as an Alternative Technique for the Socket Preservation

**DOI:** 10.1155/2022/7455050

**Published:** 2022-05-02

**Authors:** Francesco Campione, Ludovica Campione, Mario Francesco Campione, Michele Melillo, Claudia Arena, Francesc Abella, Luca Boschini

**Affiliations:** ^1^Dottor Francesco Campione Clinic, via de Caro 104 Catania, Italy; ^2^Department of Experimental and Clinical Medicine–University of Foggia, Italy; ^3^Department of Endodontics, Universitat Internacional de Catalunya, Sant Cugat del Vallès, Barcelona, Spain; ^4^Ambulatorio Polispecialistico Dottor Boschini Srl, Rimini, Italy

## Abstract

*Background and Overview*. Socket shield is a technique that allows the maintenance of tissue volumes. In the reported clinical case, the replantation of the buccal root fragment mistakenly extracted during a socket shield surgery is performed. We present a follow-up to 5 years with an unexpected healing.*Case Description*. An 88-year-old patient underwent an immediate loading implant placement associated with the replantation of the mistakenly extracted root fragment. The shaping of the fragment was performed extraorally, and the replantation was done so that the fragment protruded above the crest margin.*Conclusions and Practical Implications*. The 5-year follow-up shows an uneventful healing of the implant. At 48 months, postoperative CBCT exam reports images compatible with the regeneration of the bone over the portion of root that protruded over the margin. The outcome suggests clinical implications, as the opportunity to easily shape the fragment extraorally and replant sound portion of the root (not necessary the buccal) in buccal socket with bone defect.

## 1. Introduction

Maintenance or reconstruction of peri-implant soft tissues is still one of the main challenges for implant therapy in esthetic areas [1, 2]. Despite a high survival rate, the esthetic outcome is often compromised, especially by alveolar bone remodeling [3]. The resorption of the alveolar ridge after tooth extraction is more pronounced on the buccal than on the lingual side of the extraction socket, causing a soft tissue deficiency that can interfere with the esthetic outcome of implant supported prosthesis [4]. Several treatment approaches have been proposed in literature to maintain the tissues volume after tooth extraction [5], which is essential to preserve an esthetically pleasant soft tissue contour; among them, there are socket preservation, socket shield, gingival grafts, guided bone regeneration with membrane, and/or grafting materials [6–11]. Numerous studies show that socket preservation is a viable technique to maintain alveolar ridge volume and height. Hurzeler et al. first described the socket shield technique in 2010 [12] and the literature about this technique was reviewed by Gharpure and Bhatavadekar in 2017 [13] and by Ogawa et al. in 2021 [14]. This technique is based on the preservation of the vestibular fragment of the extracted tooth to prevent the alveolar bone resorption of the buccal side [12].

On the other hand, tooth autotransplantation is a treatment option in which a tooth is surgically moved from one site to another [15, 16]. If performed in accordance to biological principles, it ensures maintenance and regeneration of alveolar bone through physiological stimulation of periodontal ligament formation [17].

This case report shows a clinical case of a socket shield technique performed in combination with principles of teeth autotransplantation.

## 2. Case Description

**Figure 1:**
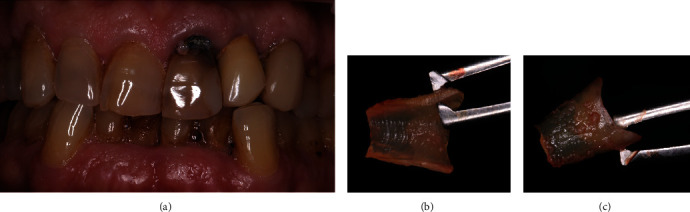
(a) Clinical aspect of the central left incisor with a great decay on the cementoenamel junction. (b and c) Portion of the root after being shaped extraorally. The periodontal ligament fibers on the root remain untouched and visible on the buccal side.

An 88-year-old man with a noncontributory medical history presented at private dental clinic in Catania, Italy, with a large cervical carious lesion on an endodontically treated upper left central incisor (Figure 1(a)). Patient requested a prompt rehabilitation of his anterior area, and therefore, extraction with placement of an immediate loaded implant was proposed. Periodontal clinical parameters recording showed no bleeding on probing. Pocket probing depth (PPD) was measured at six points for each tooth. The left central incisor showed 4 mm PPD in all the buccal points and 2 mm PPD in all the palatal points. The gum was firm and dimpled in texture; the gingival margin showed a recession of 2,5 mm due to the carious lesion affecting the cementum-enamel junction.

Cone beam computed tomography analysis was executed before surgery for the case study, to rule out the presence of periapical lesions and to evaluate the bundle bone plate volume. According to the tomography, the buccal bone was very thin, and a recession of 4 mm from the cementum-enamel junction was measured; no periradicular lesion was found.

After approval to perform immediate implant placement, the patient also gave his consent to perform the socket shield technique and signed informed consent. One hour before surgery, the patient received antibiotic prophylaxis with 2 g amoxicillin (Zimox; Pfizer). Surgery was performed under local anesthesia. The extraction was performed flapless. After crown removal with a coarse-grained diamond bur, the pulp chamber and the root canal were used as a guide to section the root in two parts following a mesiodistal direction. Then, during the luxation of the palatal fragment, the buccal portion of root was accidentally extracted. After careful examination of the avulsed fragment, the surgeons decided to replant it in the original site. Since the fragment was now extracted, the final shaping was performed extraorally. After the shaping, the root fragment measurement performed with an Iwanson caliper registered a 1 mm thickness, 4,5 mm height, and 3,5 mm width (Figures 1(b) and 1(c)). The root fragment was placed in saline solution in a sterile container.

The implant site was prepared with standard drills following the palatal bony wall as a guide. The fixture was placed 3 mm beneath the palatal level of the bone crest. A morse-taper implant (3, 8 × 10 mm, 3P Implafavourite, Torino, IT) was used. Primary implant stability assessment with resonance frequency analysis revealed an ISQ value of 70 and a Torque insertion value of 60 N/cm^2^.

After implant placement, the root membrane was repositioned about 3 mm apical to the gingival margin leaning against the buccal wall of the site and 2,5 mm above the bone crest as revealed by the postoperative computed tomography. No filling material was used to fill the void between the fragment and the implant. The stabilization of the fragment was obtained with the positioning of the provisional restoration.

The provisional restoration was obtained by rebasing the natural sectioned crown on a Ti-base abutment using a hybrid composite (Optifil – IDS Spa).

Care was taken to remove all centric and eccentric functional contacts.

**Figure 2:**
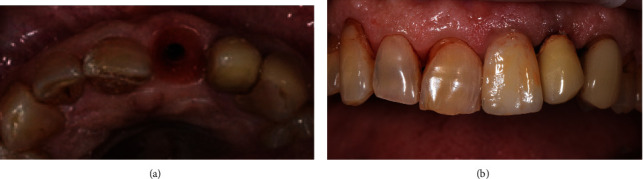
(a) Final tissue healing after 6-month follow-up. No signs of inflammation or rejection of the fragment were observed. (b) The final definitive restoration 5-year follow-up; the gingival tissues are healed and preserved.

The patient was recommended to follow a soft diet and instructed in proper oral hygiene. Postoperative evaluation was performed after 2 weeks and showed uneventful healing. Six months after implant placement, provisional restoration was removed showing the complete healing of the implant site. At this time a final optical impression was taken with CEREC CAD-CAM technology (Dentsply-Sirona), and a definitive lithium disilicate screw retained crown (IPS e.max – Ivoclar Vivadent) was realized (Figure 2(b)).

Follow-up visits were scheduled at 6 and 12 months; all the following follow-up visits were annual (the last follow-up is at 5 years without complication). At every follow-up, clinical parameters were recorded showing an uneventful healing; no clinical signs of inflammation or plaque accumulation were recorded. Intraoral pictures were taken at every follow-up examination (Figure 2(a)).

**Figure 3:**
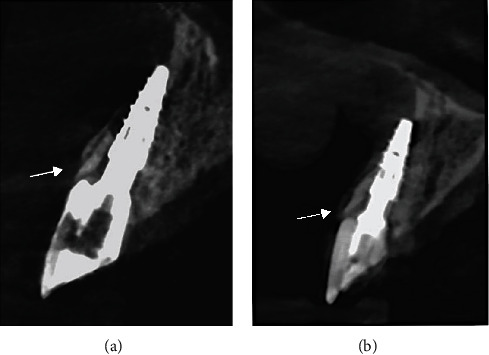
(a) At 6-month follow-up, CBCT images show the replanted root membrane in strict contact with the implant abutment and the bundle bone. The arrow indicates the portion of root not covered by bone. (b) At 48 months, CBCT images are compatible with the complete covering of the root membrane (see the arrow), as if there had been migration of the bone over all the root membrane surface.

A CBCT exam was performed at 6- and 48-month follow-up to evaluate healing or any tissue alteration (Figure 3). The root-membrane appeared well integrated in the context. Moreover, the CBCT images are suggestive of the buccal bone growth over the portion of root membrane which initially protruded beyond the margin of the bone crest (Figure 3(b)).

## 3. Discussion

Delayed implant placement has a high survival and success rate in both posterior and anterior areas [18, 19]. This kind of treatment is often more time demanding and requests multiple surgical sessions [20]. On the other hand, the resorption of the buccal bundle bone after tooth extraction and immediate implant placement can have a negative esthetic impact [21].

A buccal bone of at least 2 mm thickness seems to be required to perform a successful restoration [22].

Socket shield technique is an effective technique to prevent alveolar-ridge alterations that occur after tooth extraction reducing the possibility of a buccal bone resorption and improving the aesthetic outcome of the final restoration. This technique is also called partial extraction therapy [23] or root membrane technique [24]. It is particularly suitable for teeth in anterior areas, mainly in the maxilla, but it is achievable only in selected cases [23].

Success of this technique is mainly based on the preservation of the periodontal ligament on the vestibular portion of the root. Moreover, it is a minimally invasive procedure that reduces time of treatment and the need of soft and hard tissue grafting procedures. However, this technique is still highly operator experience sensitive [23]. Studies showed that accessing the apical aspect of the root during sectioning might be difficult, due to the poor visibility of the apical fragment, thus increasing the risk of leaving behind fragments of the root apex [25]. This might compromise the efficacy of this technique in the long term. An inappropriately trimmed root might indeed compromise the maintenance of the buccal alveolar bone volume [24]. The conventional socket shield technique can be applied only if the remaining parts of the root are periodontally healthy and if the socket is well conserved in particular on the buccal site [26].

The periodontal ligament is the success key of other well documented surgical therapies: tooth autotransplantation and replantation. Tooth autotransplantation consists in the movement of an extracted tooth from a donor site to another defined receiving socket [27–30].

This technique provides the maintenance and the regeneration of the alveolar bone volume through stimulation of viable cells of periodontal ligament preserved on the root surface [31, 32]. The same healing mechanisms were implemented in intentional replantation in which the donor and the receiving socket are the same; in fact, this technique consists in the repositioning of an extracted tooth in its socket after the extraoral management of endodontic problems [33, 34]. In this case, the vestibular fragment was intentionally replanted according to intentional replantation and transplantation protocol [33]. The rationale behind the choice of surgeons, experts in dental transplantations, is that if it is possible to extract and reposition a periodontally healthy tooth, then it will be possible to do the same with a portion of it. When the buccal root fragment dislocated and was extracted, the operators made the decision to replant the fragment rather than resorting to other surgical techniques, such as socket preservation, which would have required longer time. Having the root membrane in hand, the surgeons had the opportunity to shape the fragment more easily, taking care not to leave the apex or portion of endodontics spaces. In addition, the surgeons had the possibility to replant the fragment more coronally than the crest margin, leaving only the apical half of the fragment in contact with the wall socket; the other half of the root membrane protrude beyond the bone margin. After 48 months, CBCT images suggest a complete healing of the buccal bone; indeed, the images are compatible with the complete covering of the root membrane, as if there had been migration of the bone over all the root membrane surface. This healing is documented in tooth autotransplantation where it is possible to observe the bone regeneration of bone defect by the sound periodontal ligament present on the roots of the donor tooth [34]. The outcome was better than the surgeons would have expected, suggesting some clinical implication. The root membrane replantation technique requires some insights to be scientifically validated, unlike the socket shield technique for which the literature is abundant, but if approved, it could provide some advantages. First of all, the shaping of the root membrane can be carried out extraorally, avoiding the cited complications due to a lack of visibility during the intra oral modelling [23, 25].

In addition, the surgeon can decide to replant the root membrane in a more convenient position compared to the original.

Therefore, the root membrane replantation might allow to overcome several limitations of the conventional socket shield technique. In fact, the fundamental requirement to perform the socket shield technique is that the buccal wall of the socket is conserved. Considering the outcome of this case report, we can hypothesize the effectiveness of using a portion of periodontally healthy root, not necessarily the buccal portion, for replantation in the buccal wall of the socket even if it has a bone defect.

## 4. Conclusions

The replantation of a root membrane accidentally extracted during a socket shield technique surgery gave an unexpected outcome with the regeneration of bone over a portion of root fragment not submerged under the bone margin.

Although it is necessary to scientifically validate this technique, it is possible to hypothesize some clinical implications:

1. In case of involuntary extraction of the buccal fragment during a socket shield surgery, it is possible to replant the fragment without diverting to other surgical techniques
2. The possibility of extraoral shaping making the procedure easier than performing it intraorally
3. The possibility of replantation of root fragment other than the buccal even in the case of a buccal bone defect of the socket, favoring its regeneration
